# Exploring the Mechanism of Adjuvant Treatment of Glioblastoma Using Temozolomide and Metformin

**DOI:** 10.3390/ijms23158171

**Published:** 2022-07-25

**Authors:** Shao-Wei Feng, Pei-Chi Chang, Hsuan-Yu Chen, Dueng-Yuan Hueng, Yao-Feng Li, Shih-Ming Huang

**Affiliations:** 1 Department of Neurologic Surgery, Tri-Service General Hospital, National Defense Medical Center, Taipei 114, Taiwan; heisenber0930@gmail.com (S.-W.F.); hondy2195@gmail.com (D.-Y.H.); 2 Graduate Institute of Life Sciences, National Defense Medical Center, Taipei 114, Taiwan; brooke19961013@gmail.com; 3 Department of Biochemistry, National Defense Medical Center, Taipei 114, Taiwan; 4 Department of Radiology, Tri-Service General Hospital, National Defense Medical Center, Taipei 114, Taiwan; penguin0916@livermail.tw; 5 Department of Pathology, Tri-Service General Hospital, National Defense Medical Center, Taipei 114, Taiwan

**Keywords:** metformin, glioma, glioblastoma, adjuvant treatment, temozolomide, O^6^-methylguanine-DNA methyltransferase

## Abstract

Glioblastoma is the most frequent and lethal primary central nervous system tumor in adults, accounting for around 15% of intracranial neoplasms and 40–50% of all primary malignant brain tumors, with an annual incidence of 3–6 cases per 100,000 population. Despite maximum treatment, patients only have a median survival time of 15 months. Metformin is a biguanide drug utilized as the first-line medication in treating type 2 diabetes. Recently, researchers have noticed that metformin can contribute to antineoplastic activity. The objective of this study is to investigate the mechanism of metformin as a potential adjuvant treatment drug in glioblastoma. Glioblastoma cell lines U87MG, LNZ308, and LN229 were treated with metformin, and several cellular functions and metabolic states were evaluated. First, the proliferation capability was investigated using the MTS assay and BrdU assay, while cell apoptosis was evaluated using the annexin V assay. Next, a wound-healing assay and mesenchymal biomarkers (N-cadherin, vimentin, and Twist) were used to detect the cell migration ability and epithelial–mesenchymal transition (EMT) status of tumor cells. Gene set enrichment analysis (GSEA) was applied to the transcriptome of the metformin-treated glioblastoma cell line. Then, DCFH-DA and MitoSOX Red dyes were used to quantify reactive oxygen species (ROS) in the cytosol and mitochondria. JC-1 dye and Western blotting analysis were used to evaluate mitochondrial membrane potential and biogenesis. In addition, the combinatory effect of temozolomide (TMZ) with metformin treatment was assessed by combination index analysis. Metformin could decrease cell viability, proliferation, and migration, increase cell apoptosis, and disrupt EMT in all three glioblastoma cell lines. The GSEA study highlighted increased ROS and hypoxia in the metformin-treated glioblastoma cells. Metformin increased ROS production, impaired mitochondrial membrane potential, and reduced mitochondrial biogenesis. The combined treatment of metformin and TMZ had U87 as synergistic, LNZ308 as antagonistic, and LN229 as additive. Metformin alone or combined with TMZ could suppress mitochondrial transcription factor A, Twist, and O^6^-methylguanine-DNA methyltransferase (MGMT) proteins in TMZ-resistant LN229 cells. In conclusion, our study showed that metformin decreased metabolic activity, proliferation, migration, mitochondrial biogenesis, and mitochondrial membrane potential and increased apoptosis and ROS in some glioblastoma cells. The sensitivity of the TMZ-resistant glioblastoma cell line to metformin might be mediated via the suppression of mitochondrial biogenesis, EMT, and MGMT expression. Our work provides new insights into the choice of adjuvant agents in TMZ-resistant GBM therapy.

## 1. Introduction

Brain tumors are responsible for substantial morbidity and mortality worldwide. Gliomas are the most common central nervous system (CNS) malignancies, accounting for one-quarter of all CNS neoplasms. The most lethal glioma is *isocitrate dehydrogenase* (*IDH*) wild-type glioblastomas, World Health Organization (WHO) grade 4 [1]. Since the revision of the updated edition of the WHO classification published in 2016 [2], molecular information has played a critical role in prognosis prediction and treatment, including *IDH* [3,4], 1p19q [2], *epidermal growth factor receptor amplification* [5], chromosome 7 gain and chromosome 10 alterations [5], *telomerase reverse transcriptase* promoter mutation [5], *cyclin-dependent kinase inhibitor 2A/B* homozygous deletion [6], and *O^6^-methylguanine-DNA methyltransferase* (*MGMT*) methylation status [7]. However, despite these advances in molecular stratification, the therapeutic choice is still limited because of the considerable blood–brain barrier; therefore, there are a few efficient drugs available against this deadly neoplasm. Temozolomide (TMZ), an oral alkylating agent, has been widely used as a first-line chemotherapeutic agent since 2005 [8]. However, drug resistance often occurs because of the loss of *MGMT* methylation or the base excision repair mechanism [9]. Hence, the overall survival among patients with glioblastoma remains poor at 14.6 months. Therefore, there is a need to identify new biomarkers and treatments that improve patient outcomes.

Metformin is a biguanide drug utilized as the first-line medication in treating type 2 diabetes. It represses gluconeogenesis in the liver, stimulates peripheral cells to insulin, enhances glucose uptake, inhibits mitochondrial respiration, and decreases glucose absorption by the gastrointestinal tract [10,11,12,13]. Metformin is a generally safe drug under careful monitoring (i.e., outpatient clinic follow-up, renal function, hemoglobin A1c, etc.); it has been used daily in millions of patients for a long time. Although the most critical complication of metformin is lactic acidosis, its occurrence in patients is unusual [14,15,16]. Repositioning metformin for cancer therapy or prevention is also applicable to buformin and phenformin, both of which are similar in function to metformin in current cancer treatment. Higher doses of biguanides might apply a direct antitumor impact at doses lower than those used for antidiabetic effects. A range of doses exists for biguanides to serve as diabeto-biguanides (for type 2 diabetes mellitus) or onco-biguanides (for cancers) [17]. Their potential influence on numerous cancers such as leukemia [18], colon cancer [19], melanoma [20,21,22], breast cancer [23], prostate cancer [24], pancreatic cancer [25,26], endometrial carcinoma [27], and lung cancer [28] has also been investigated. Metformin has also been associated with better overall and progression-free survival in high-grade glioma patients [29]. However, there is still insufficient evidence underlying the therapeutic role of metformin in glioblastoma.

The present study aims to investigate the mechanism of metformin as a potential treatment drug in glioblastoma. Furthermore, metformin might act via potential mechanisms to overcome resistance to current TMZ treatment. Our work provides new insight into the choice of therapeutic agents and TMZ resistance in glioblastoma treatment.

## 2. Results

### 2.1. Metformin-Decreased Cell Viability and Proliferation in a Dose-Dependent Manner in Three GBM Cell Lines

**Figure 1:**
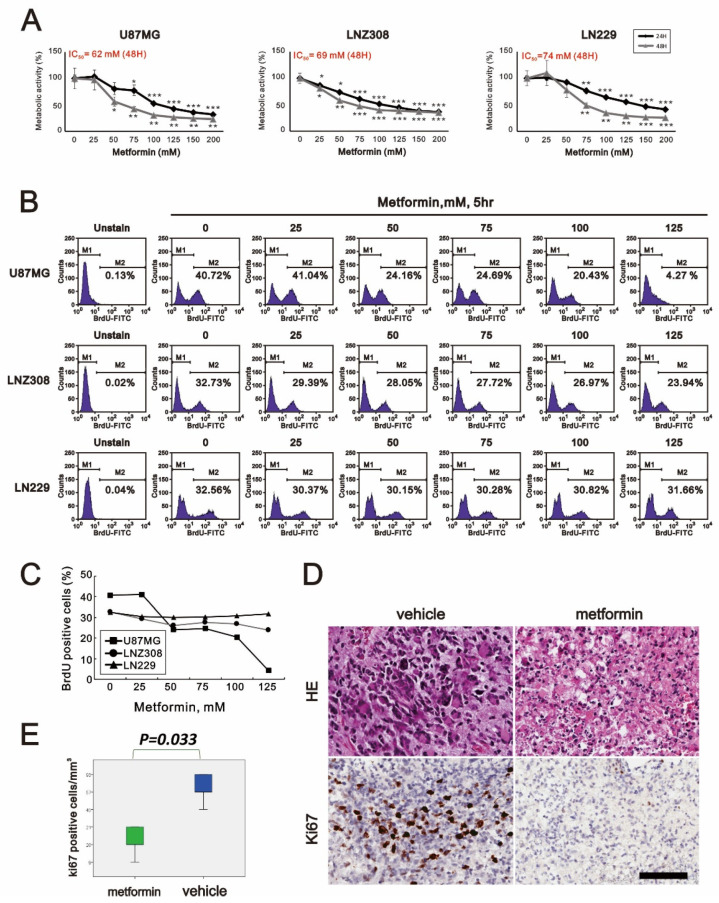
The effects of metformin on cell viability and proliferation in GBM. (**A**) U87MG, LNZ308, and LN229 cells were treated with the indicated metformin concentrations for 24 and 48 h. Metabolic activity was measured using MTS assays. * *p* < 0.05, ** *p* < 0.01, and *** *p* < 0.001. (**B**) U87MG, LNZ308, and LN229 cells were treated with the indicated metformin concentrations for 5 h. They were subjected to BrdU proliferation analysis and (**C**) their quantification. (**D**,**E**) Sliced GBM tissue was treated with vehicle (control) or 100 mM metformin for 3 days and statistically analyzed using Student’s *t*-test (significance was set at *p* < 0.05). Scale bar is 50 µm.

Metformin has been reported to be an antitumor agent in many cancers. This study applied metformin to address its antitumor mechanisms in three GBM cell lines, U87MG, LNZ308, and LN229. First, GBM cells were supplemented with indicated concentrations of metformin for 24 and 48 h to analyze its cytotoxic effect on U87MG, LNZ308, and LN229 cells. We found that the cell viability was suppressed in a metformin dose-dependent manner (Figure 1A). For the 48 h experiment, the IC_50_ values of U87MG, LNZ308, and LN229 cell lines were 62, 69, and 74 mM, respectively (Figure 1A). To further determine if the decrease in cell viability was relevant to cellular proliferation, we used the BrdU assay to examine the cell proliferation status with the indicated metformin concentrations (Figure 1B,C). In the U87MG cells, the BrdU of 125 mM metformin-treated cells dropped to 4.27% from the original 40.72%. For the LNZ308 cells, it fell to 23.94% compared to the control value of 32.73%. Interestingly, it only showed a slight decrease from 32.56% to 31.66% in LN229 cells. In these experiments, we noted that metformin could impair glioma cell viability and was relevant to the suppression of cell proliferation. We further applied 100 mM metformin in the organotypic slice culture of GBM. Furthermore, the proliferative index, Ki-67, also significantly decreased when supplemented with metformin compared to the control (Figure 1D,E).

### 2.2. Metformin Induced Apoptosis in All Three GBM Cell Lines

**Figure 2:**
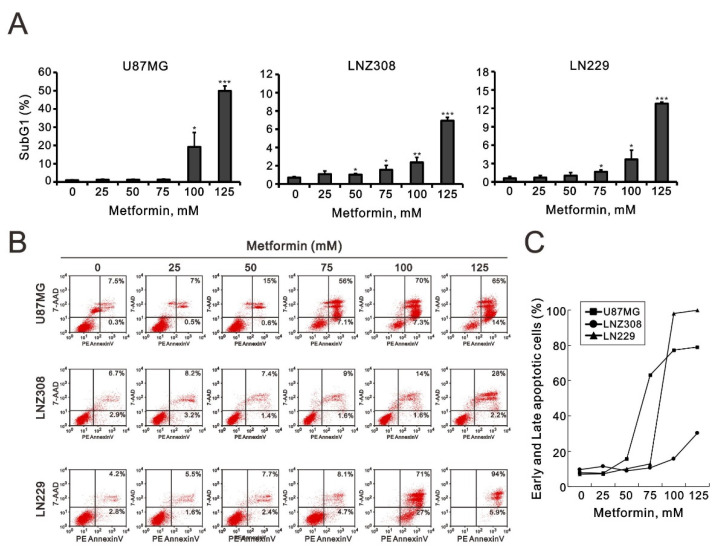
The effects of metformin on the cell-cycle profile and cellular apoptosis in GBM cell lines. (**A**) U87MG, LNZ308, and LN229 cells were treated with the indicated metformin concentrations for 24 h. The population of cells in the sub-G1 phase of the cell cycle was measured using flow cytometry analysis and statistically plotted. * *p* < 0.05, ** *p* < 0.01, and *** *p* < 0.001. (**B**) U87MG, LNZ308, and LN229 cells were treated with the indicated metformin concentrations for 24 h. Cells were stained with PE–annexin V and 7-AAD and were measured using flow cytometry analysis. Their quantification of early and late apoptotic cells showed as (**C**).

The cytotoxic effect of metformin might be due to the suppression of cellular proliferation and the induction of apoptosis. As shown in Figure 1, metformin altered cellular proliferation in U87MG and LNZ308 cells. Hence, we evaluated apoptosis according to the cell-cycle profile and annexin V staining using flow cytometry analysis. We found that the proportion of cells in the sub-G1 phase increased dramatically in a metformin dose-dependent manner in U87MG, LNZ308, and LN229 cells (Figure 2A). To further investigate why the percentage of cells in the sub-G1 phase increased, we examined the cellular apoptosis using annexin V staining. We noticed a significant dose-dependent increase in late-stage apoptosis in U87MG, LNZ308, and LN229 cells (Figure 2B,C).

### 2.3. Metformin Inhibited Cell Migration and Disrupted Epithelial–Mesenchymal Transition in GBM Cells

**Figure 3:**
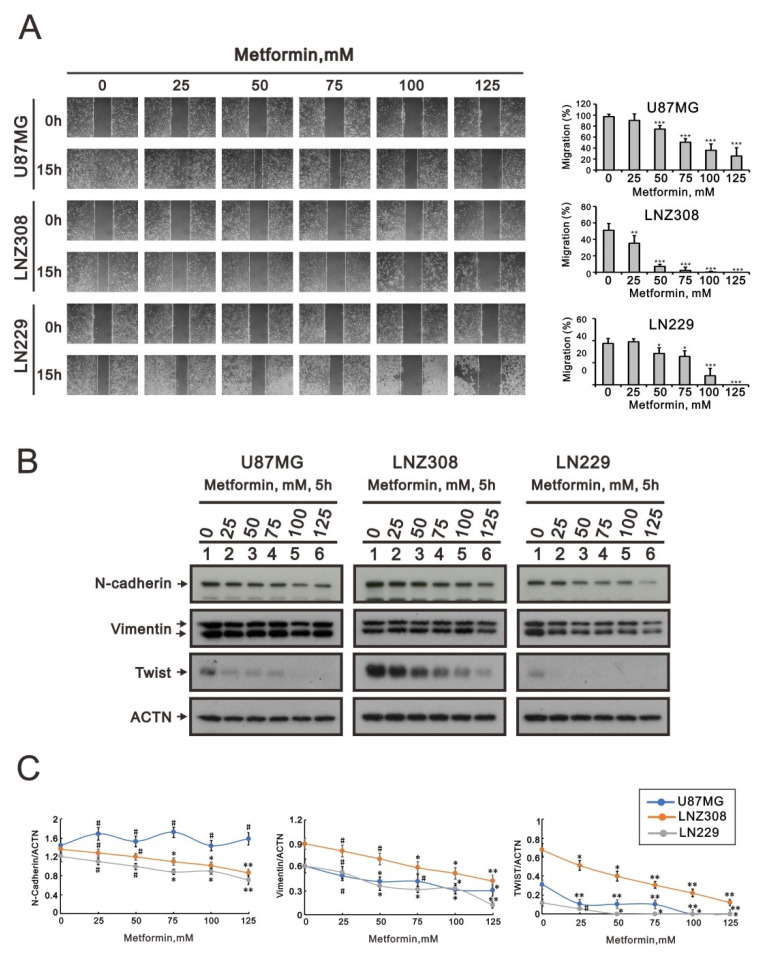
The effects of metformin on cell migration and EMT in GBM cell lines. (**A**) U87MG, LNZ308, and LN229 cells were treated with the indicated metformin concentrations. After 15 h, the cell migration was measured; * *p* < 0.05 and *** *p* < 0.001. (**B**) U87MG, LNZ308, and LN229 cells were treated with the indicated metformin concentrations for 5 h. Cell lysates were subjected to Western blot analysis using antibodies against the indicated proteins; ACTN was used as the protein-loading control. The protein bands were quantified through pixel density scanning and evaluated using ImageJ, version 1.44a (http://imagej.nih.gov/ij/) (accessed on 15 July 2022). (**C**) The ratios of N-cadherin/ACTN, Vimentin/ACTN, and TWIST/ACTN were plotted in the U87MG, LNZ308, and LN229 cells. The results are representative of three independent experiments. # *p* > 0.05, * *p* < 0.05, ** *p* < 0.01, and *** *p* < 0.001.

To further understand the relationship between metformin and alterations in cellular function, such as tumor migration ability and epithelial–mesenchymal transition (EMT), wound-healing assays were carried out with the U87MG, LNZ308, and LN229 cell lines. According to the results, 15 h after wounding, the migration area was significantly smaller in cultures with a higher metformin treatment concentration (Figure 3A). Next, we used three mesenchymal biomarkers (N-cadherin, vimentin, and Twist) to evaluate the EMT status of these tumor cells. The results showed a significant decrease in N-cadherin, vimentin, and Twist in some metformin-treated cells (Figure 3B,C). These experiments suggest that the reduction in EMT after metformin supplementation may be attributed to the reduction in migration.

### 2.4. The Transcriptome of the Metformin-Treated Group Revealed Distinct Signatures Compared to the Control with an Associated Increase in Reactive Oxygen Species and Hypoxia

**Figure 4:**
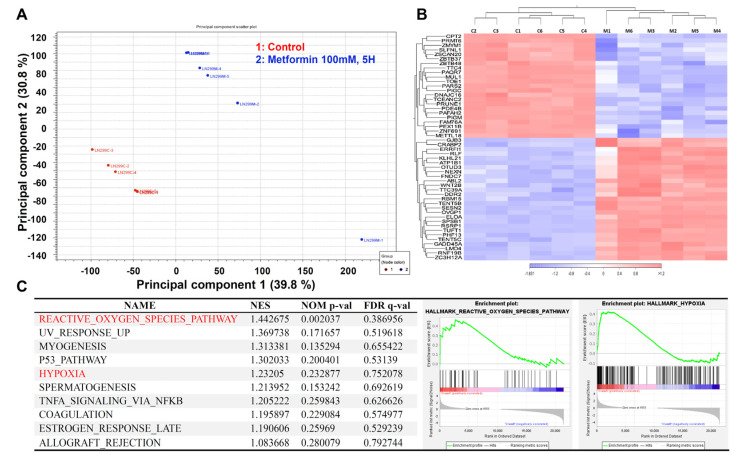
The effect of metformin on the transcriptome of LN229 cells. LN229 cells were treated with vehicle or 100 mM metformin for 5 h. Total RNA was extracted for RNA sequencing analysis. (**A**) PCA revealing different clusters of metformin-treated and control cells. (**B**) Heatmap showing the top differentially expressed genes in both groups. (**C**) GSEA study highlighting the increase in ROS and hypoxia in metformin-treated LN229 cells.

Having determined the effects of metformin supplementation on GBM cell viability, proliferation, apoptosis, migration, and EMT status, we aimed to investigate the functional pathways mediating this activity. We compared LN229 cells treated with 100 mM metformin to the control group (*n* = 6 in each group) for RNA-sequencing analysis. In the principal component analysis (PCA), two separate clusters were identified (Figure 4A), matching perfectly with the initial group separation (control vs. metformin-treated cells). Then, each group’s top differentially expressed genes were determined and demonstrated as a heatmap (Figure 4B). Next, the transcriptome data were uploaded into the GSEA platform to investigate the list of altered pathways. According to the top 10 lists, reactive oxygen species (ROS) and hypoxia were identified as the main effects of metformin supplementation on LN229 GBM cells (Figure 4C).

### 2.5. Metformin Increased ROS Production, Impaired Mitochondrial Membrane Potential (MMP), and Reduced Mitochondrial Biogenesis in GBM Cells

**Figure 5:**
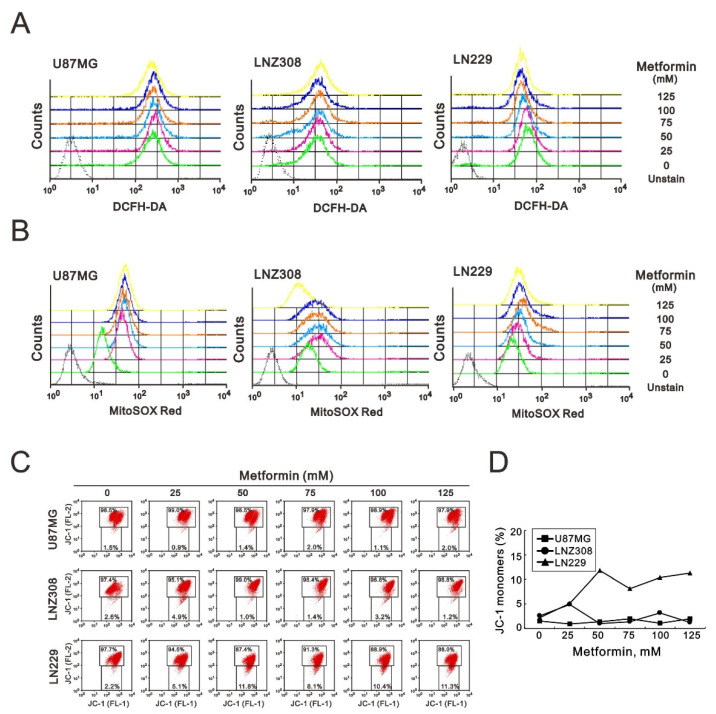
The effects of metformin on cytosolic ROS, mitochondrial ROS, and mitochondrial membrane potential in GBM cells. U87MG, LNZ308, and LN229 cells were treated with the indicated metformin concentrations. After 3 h, (**A**) the cytosolic ROS was measured with DCFH-DA, and (**B**) the mitochondrial ROS was measured with the MitoSOX Red kit using flow cytometry analysis. (**C**) U87MG, LNZ308, and LN229 cells were treated with the indicated metformin concentrations for 5 h. The integrity of the mitochondrial membrane potential was measured with JC-1 dye using flow cytometry analysis. Their quantification of monomer (FL-1, green fluorescence) form of the dye showed as (**D**).

According to the GSEA, metformin-treated cells significantly increased the pathway of ROS generation. Next, we quantified ROS in the cytosol and mitochondria using DCFH-DA and MitoSOX Red dyes, respectively. We found that metformin induced cytosolic ROS production in U87MG and LNZ308 cell lines, but its effect was not obvious in LN229 cells (Figure 5A). Metformin induced mitochondrial ROS production in U87MG, LNZ308, and LN229 cells, whereas the highest concentration (125 mM) of metformin had no effect in LNZ308 cells (Figure 5B).

Metformin is a well-known inhibitor of complex I of the electron transport chain in mitochondria. Hence, we further examined the effect of metformin on MMP using JC-1 dye in GBM cells. Our JC-1 data showed that metformin only disrupted the MMP in LN229 cells (Figure 5C,D).

**Figure 6:**
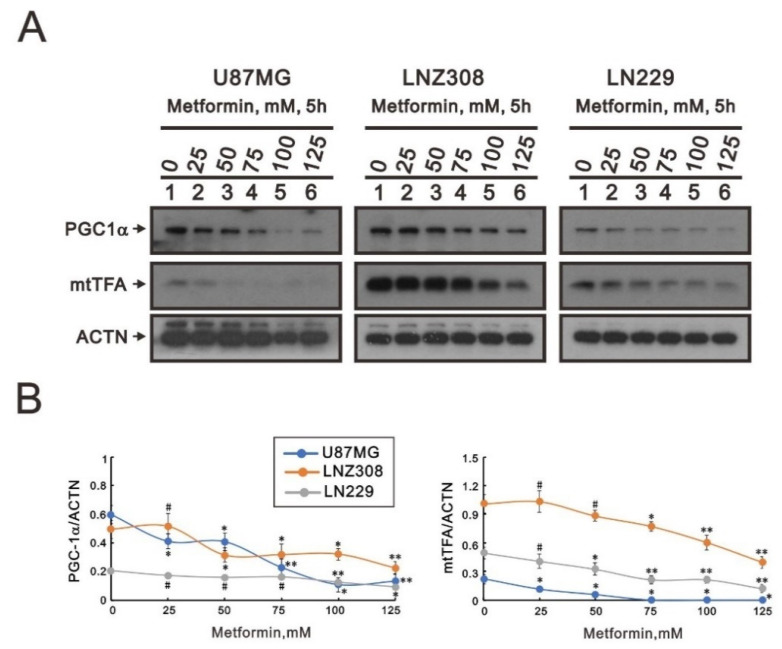
The effects of metformin on mitochondrial biogenesis in GBM cells. U87MG, LNZ308, and LN229 cells were treated with the indicated metformin concentrations. After 5 h, cell lysates were collected and subjected to (**A**) Western blotting analysis using antibodies against PGC-1α and mtTFA. ACTN was used as the protein-loading control. The protein bands were quantified through pixel density scanning and evaluated using ImageJ, version 1.44a (http://imagej.nih.gov/ij/) (accessed on 15 July 2022). (**B**) The ratios of PGC-1α/ACTN and mtTFA/ACTN were plotted in the U87MG, LNZ308, and LN229 cells. The results are representative of three independent experiments. # *p* > 0.05, * *p* < 0.05, and ** *p* < 0.01.

Peroxisome proliferator-activated receptor γ coactivator-1α (PGC-1α) and mitochondrial transcription factor A (mtTFA) are two key mitochondrial respiratory and biogenic factors for mitochondrial respiratory function [30,31]. Our Western blotting data (Figure 6A,B) showed that metformin tended to significantly decrease levels of both PGC-1α and mtTFA proteins in U87MG, LNZ308, and LN229 cells, suggesting that ROS production, membrane potential, and biogenesis might be disrupted in the mitochondria of metformin-treated GBM cells.

### 2.6. The Combinatory Effect of TMZ with Metformin on GBM Cells

**Figure 7:**
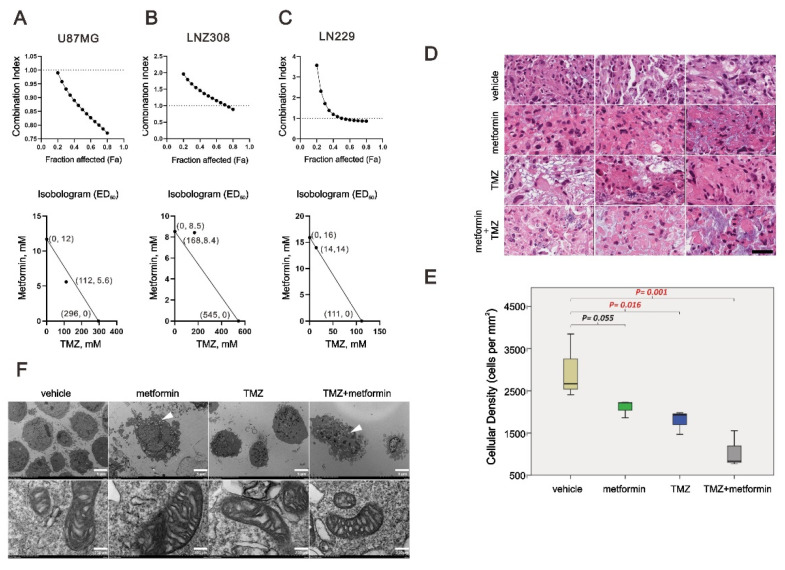
The combination index of TMZ and metformin in GBM cells. U87MG, LNZ308, and LN229 cells were treated with 0, 15.625, 31.25, 62.5, 125, 250, 500, 1000, 2000 m, and 4000 µM TMZ and 0, 3.125, 6.25, 12.5, 25, 50, 100, and 200 mM metformin for 72 h. Metabolic activity was measured using MTS assays. The combination index of TMZ plus metformin in U87MG (**A**), LNZ308 (**B**), and LN229 (**C**) cells. Isobolograms (ED_50_) of TMZ or metformin were calculated using CalcuSyn software. (**D**,**E**) Patient-derived organoid culture was separated into four groups with different drug supplements, including Vehicle, 50 mM metformin, 100 µM TMZ, and combined 50 mM metformin with 100 µM TMZ. Scale bar = 50 μm. (**F**) LN229 cells were treated with 50 mM metformin, 12.5 µM TMZ, or their combination. After 48 h, the detailed mitochondrial structure of LN229 cells was investigated using the H-7500 TEM. Scale bar = 5 μm (top) and 250 μm (bottom).

TMZ is the only chemotherapeutic agent available for GBM treatment. We further examined whether metformin had a synergistic effect with TMZ on U87MG, LNZ308, and LN229 cells using combination index analysis. A combination index score <1 indicates a synergistic effect, whereas a score >1 indicates an antagonistic effect. The results showed that U87 was synergistic, LNZ308 was antagonistic, and LN229 was additive. U87MG, LNZ308, and LN229 cells dramatically reduced the dose of TMZ from 296, 545, and 111 μM to 112, 168, and 14 μM when metformin was supplemented at 5.6, 8.4, and 14 mM, respectively (Figure 7A–C). Patient-derived organoid culture was separated into four groups with different drug supplements, including vehicle, 50 mM metformin, 100 μM TMZ, and combined 50 mM metformin with 100 μM TMZ. The tumor cells in the control groups showed healthy tumor nuclei showing marked hyperchromatic. On the contrary, the tumor showed marked cytoplasm vacuoles and eosinophilia with the trend of decreasing tumor cells (*p* < 0.05) when supplemented with either metformin, TMZ, or a combination. In the comparison, we noticed that the group combined with metformin and TMZ showed the most apparent changes in the morphology and number of tumor cells (Figure 7D,E). We further examined the effect of metformin, TMZ, and their combination on the mitochondria of LN229 cells using a transmission electron microscope (TEM). We observed that the cancer cells revealed marked cytoplasm vesicles in the metformin treatment groups, whether alone or combined with TMZ (Figure 7E, white arrowhead). In contrast, no dramatic increase in cytoplasm vesicles was observed in the TMZ-only treatment group. However, there was no apparent difference in terms of cristae among these groups.

### 2.7. The Effect of Metformin on Mitochondrial Biogenesis and EMT in TMZ-Sensitive and TMZ-Resistant (TMZ-R) Glioma Cells

**Figure 8:**
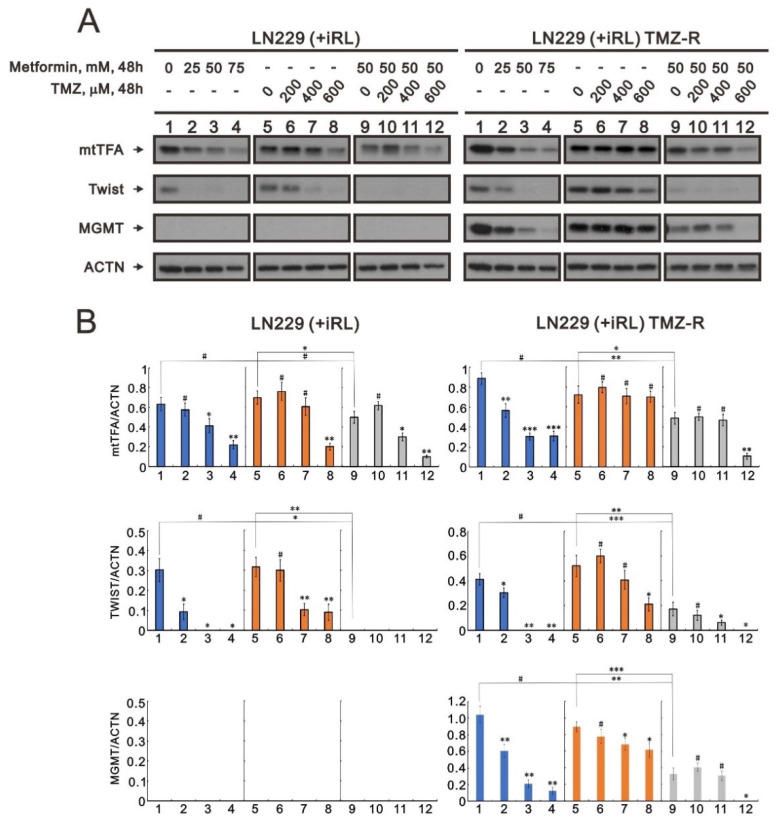
The effects of metformin, TMZ, and their combination on MGMT proteins in TMZ-resistant LN229 cells. LN229 parent and resistant cells, LN229 (+IRL) and LN229 (+IRL) TMZ-R cells, were treated with the indicated concentrations of metformin, TMZ, and their combination. (**A**) After 48 h, cell lysates were collected and subjected to Western blotting analysis using antibodies against mtTFA, Twist, and MGMT. ACTN was used as the protein-loading control. The protein bands were quantified through pixel density scanning and evaluated using ImageJ, version 1.44a (http://imagej.nih.gov/ij/) (accessed on 15 July 2022). (**B**) The ratios of mtTFA/ACTN, TWIST/ACTN, and MGMT/ACTN were plotted in the LN229 (+IRL) and LN229 (+IRL) TMZ-R cells. The results are representative of three independent experiments. # *p* > 0.05, * *p* < 0.05, ** *p* < 0.01, and *** *p* < 0.001.

Our data showed that metformin suppressed mitochondrial biogenesis and EMT in GBM cells. We further addressed whether metformin could suppress mitochondrial biogenesis and EMT in TMZ-resistant GBM cells, i.e., the LN229 (+IRL) TMZ-R cell line. The induction of the MGMT protein is a well-known mechanism for the failure of TMZ treatment in GBM patients. Our LN229 (+IRL) TMZ-R cells exhibited an induction of MGMT proteins compared with LN229 (+IRL) parent cells (Figure 8A,B). In LN229 (+IRL) TMZ-R cells, metformin alone or combined with TMZ could significantly suppress the induction of MGMT proteins in a dose-dependent manner. We observed similar suppressive patterns for mtTFA and Twist in LN229 (+IRL) parent cells with LN229 (+IRL) TMZ-R cells. Our data support that metformin might overcome TMZ resistance in GBM treatment.

## 3. Discussion

Metformin was observed to inhibit the cell growth of various tumors such as lung cancer, breast cancer, colon cancer, prostate cancer, endometrial carcinoma, melanoma, leukemia, and pancreatic cancer [18,19,20,21,22,23,24,25,26,27,28]. Our experiment showed that metformin affected cell viability and proliferation to different degrees in glioblastoma cell lines U87MG, LNZ-308, and LN-299. Ki-67 is a nuclear antigen that indicates cell proliferation, and it is a prognostic factor in gliomas [32]. Our data showed a decrease in Ki-67-positive cells in the metformin-treated glioblastoma tissue. This is the first time that metformin has been shown to reduce the proliferation index, Ki-67, on the human organotypic slice culture platform in glioma.

Metformin can induce cell-cycle arrest in different cell-cycle phases in various cancer cells. In human osteosarcoma, metformin treatment inhibits cell proliferation by arresting progression in the G2/M phase [33]. Metformin induces G0/G1 phase cell-cycle arrest in myeloma and colon cancer [34,35]. Metformin can also affect apoptosis by increasing caspase 3 activity in human glioblastoma cells [36]. We noticed a significant dose-dependent increase in total apoptosis and late-stage apoptosis in three glioblastoma cell lines. Nevertheless, early-stage apoptosis was observed in U87MG and LN229 cells, but not LNZ308 cells.

Accordingly, the effects of metformin on different glioblastoma cell lines were inconsistent. Glioblastoma is highly infiltrative and invasive in brain structures via nearby rearrangement of the extracellular matrix [37]. Thus, glioblastoma is impossible to remove completely, and it has a high rate of recurrence. Metformin was found to decrease cell migration and invasion by deregulating miRNAs in pancreatic cancer cells [38]. Additionally, metformin could suppress human HEK/TL4 cell migration by downregulating interleukin-8 [39]. Metformin also inhibited migration in hepatocellular carcinoma by activating the AMPK pathway [40]. The migration ability of U87MG cells did not change significantly under low-dose metformin treatment but began to decline at 50 mM with statistical significance. In contrast, the migration of LNZ308 cells decreased dose-dependently with statistical significance. Under low-dose metformin treatment, the migratory capacity of LN229 cells was not affected by metformin but began to decline at 50 mM with statistical significance. According to the above results, metformin could affect the migration ability of glioblastoma cells, but the effect on each glioblastoma cell was different. Among them, the migration ability of LNZ308 cells was most easily affected by metformin, whereas LN229 was less susceptible. Migration ability is based on EM. Glioblastoma’s centrifugal migration happens in the EMT state. EMT is a characteristic of glioblastoma without treatment, but it can also be induced by surgical trauma, chemotherapy, and radiotherapy [41]. This study found that N-cadherin, vimentin, and Twist were downregulated in all three glioblastoma cell lines (U87MG, LNZ308, and LN229) with the increase in the metformin dose, indicating that metformin could reduce glioblastoma migration and invasion by inhibiting EMT.

According to the GSEA, the metformin-treated glioblastoma cells significantly increased the pathway of ROS generation. In a previous study, metformin was shown to inhibit colorectal cancer cell proliferation by increasing ROS generation and enhancing sensitivity to cisplatin [35,42]. In contrast, metformin could induce human breast cancer cell apoptosis by reducing ROS production [43]. Theoretically, metformin inhibits SOD (superoxide dismutase) activity and excessive ROS formation in the mitochondria [36]. Our data showed that ROS increased in the cytoplasm and mitochondria in U87MG cells. In LNZ308 cells, cytoplasmic ROS increased with an increasing dose of metformin, while mitochondrial ROS increased in a dose-dependent manner at low doses. When the dose increased to 125 mM, ROS began to exhibit a downward trend. In LN229 cells, cytoplasmic ROS production decreased at low doses of metformin but started to increase at 125 mM. In contrast, mitochondrial ROS was reduced at low doses, before increasing dose-dependently and then beginning to decline at 100 mM. Therefore, ROS generation that is due to metformin treatment was inconsistent across the different glioblastoma cell lines.

GSEA also identified an increase in the hypoxia pathway in metformin-treated glioblastoma cells. The mitochondria are critical to regulating cellular energy and metabolism, and they play a crucial role in maintaining cancer cell growth and survival. The membrane potential gradient of mitochondria is derived from the electron transport chain responsible for ATP synthesis [44]. The mitochondrial membrane potential declines when apoptosis occurs because of the release of cytochrome c from the mitochondria [45]. However, our data showed that metformin reduced the MMP gradient only in LN299 cells.

Mitochondria are critical mediators of tumorigenesis, as they are integral in stress sensing, which allows tumor adaptation to the environment [46]. Mitochondrial biogenesis involves the synthesis of new mitochondrial DNA (mtDNA), protein, and membrane, resulting in an increase in mitochondrial number and size. PGC-1α, a co-transcriptional regulation factor, regulates mitochondrial biogenesis via activating downstream transcription factors, including nuclear respiratory factors 1 and 2, and mtTFA. mtDNA is transcribed by the mitochondrial RNA (mtRNA) polymerase (POLRMT), which is enhanced by mtTFA. mtTFA facilitates the unwinding and flexing of mtRNA, ensuring the binding of POLRMT to the mtDNA promoters [30,31].

Metformin activates AMP-activated protein kinase (AMPK), which is an essential factor for PGC-1α post-translational modification in most tissue. PGC-1α phosphorylation is an element of mitochondrial biogenesis that is regulated only by AMPK [47]. High PGC-1α expression is correlated with metastasis and poor prognosis in breast and prostate cancer [48]. Our data showed that metformin could suppress PGC-1α and mtTFA expression, indicating that metformin could reduce mitochondrial biogenesis in all three glioblastoma cell lines.

Despite maximum treatment of glioblastoma, including surgical treatment, radiotherapy, and chemotherapy, patients only have a median survival time of 15 months [49]. The most common reason why glioblastoma treatment fails is TMZ resistance. MGMT plays the most important role in TMZ chemoresistance. MGMT can repair the main cytotoxic base pair O^6^-methylguanine, which is replaced by alkylating agent TMZ [9]. Metformin combined with chemotherapeutic agents can inhibit tumor metastasis via synergic effects in digestive system cancers, reproductive system cancers, prostate cancer, breast cancer, and lung cancer [50]. When evaluating brain drugs, the blood–brain barrier should be considered, as it can intercept most pharmaceuticals. Metformin can rapidly penetrate the blood–brain barrier to protect neurons [51]. Our data showed that metformin could suppress MGMT expression in TMZ-resistant glioblastoma cells (LN229 (+IRL) TMZ-R), suggesting that it can be repurposed to overcome TMZ resistance in glioblastoma tissue.

In addition to preclinical studies demonstrating that metformin could decrease glioblastoma cell proliferation and tumor growth both in vitro and in vivo [52], in 2019, Seliger et al. published a retrospective report showing that patients with glioma grade III had better overall and progression-free survival under metformin treatment. This could be related to WHO grade III gliomas having a higher percentage of IDH mutations, which may increase sensitivity to metformin [29]. From the comparison, all three cell lines are characteristics and unique. Briefly, in the new WHO classification system, the U87MG should be categorized as IDH mutant astrocytoma, Grade 4, while the others belong to IDH wild-type glioblastoma, Grade 4 (Table 1). Another independent article also demonstrated that gliomas with IDH mutations are metformin-hypersensitive [53]. These independent studies remarkably correlated our findings that the IDH mutant astrocytoma, U87MG, was much more sensitive to metformin than the other two cell lines. Furthermore, the differences between LNZ308 and LN229 include *p53*, *PTEN*, *p16*, and *p14* status, which might be the factors that caused the variable results between them. Cells with mutated IDH are deficient in reductive glutamine anaplerosis. Moreover, metformin can decrease the anaplerotic entry of glutamine into the tricarboxylic acid cycle by inhibiting oxidative glutamine anaplerosis. In consequence, these two conditions can lead to disorders of cell metabolism, reducing their survival [54]. Our findings indicate that metformin is worthy of further clinical study.

## 4. Materials and Methods

### 4.1. Cell Culture and Chemicals

U87MG, LNZ308, and LN229 cells were cultured in Dulbecco’s modified Eagle’s medium (DMEM) containing 10% fetal bovine serum (FBS) and 1% penicillin–streptomycin (Invitrogen, Waltham, MA, USA) at 37 °C and 5% CO_2_. Metformin, TMZ, and 2′,7-dichlorofluorescein diacetate (DCFH-DA) were obtained from Sigma-Aldrich (St. Louis, MO, USA).

### 4.2. Metabolic Activity Analysis and the Combination Index

In summary, the cells were seeded onto 96-well plates and cultured for 24 h in the presence of the indicated drugs. Thermo’s Multiskan EX plate reader (Thermo Fisher Scientific, Waltham, MA, USA) was used to measure the absorbances at 490 nm after the cells were incubated with MTS solution (20 μL/well) for 2 h at 37 °C. MTS assay was performed using the CellTiter 96 Aqueous One Solution Cell Proliferation Assay Kit (Promega, Madison, WI, USA). To calculate the relative metabolism, the absorbance ratio between cells treated with drugs and untreated controls was multiplied by 100. To generate the isobologram, we utilized CalcuSyn (Biosoft, Cambridge, UK) to calculate the combination index (CI). As a rule of thumb, CI values <1 represent synergistic combination effects, whereas CI values > 1 represent antagonistic combination effects [55].

### 4.3. Fluorescence-Activated Cell Sorting (FACS), Cell-Cycle Profiling, Cellular Proliferation, and Analyses

We performed BrdU/7-AAD analysis using the FITC BrdU Flow Kit (BD Biosciences, San Jose, CA, USA) to determine the cell-cycle profile and cellular proliferation according to the manufacturer’s instructions and as previously described [56,57]. We measured cell-cycle profiles using FACS to determine the quantity of DNA in the cells. We fixed the cells in 70% ice-cold ethanol, stored them at −30 °C overnight, and washed them twice with ice-cold water, phosphate-buffered saline (PBS) supplemented with 1% FBS, and then stained with 7-AAD. The percentage of sub-G1 populations was determined using flow cytometry. To determine the incidence of apoptosis, we used BD Biosciences’ PE-annexin V Apoptosis Detection Kit. Apoptotic cell analysis was conducted using flow cytometry by using FACSCalibur flow cytometers (BD Biosciences), and the results were analyzed using Cell Quest Pro (BD Biosciences).

### 4.4. Cytosolic and Mitochondrial Reactive Oxygen Species (ROS) Assays

To detect the production of cytosolic ROS, we plated cells in six-well plates and treated them with metformin. After 3 h of drug treatment, living cells were stained with 10 μM DCFH-DA (Sigma-Aldrich) and incubated at 37 °C for 10 min. Stained cells were determined using flow cytometry. The fluorescent marker MitoSOX™ Red (Invitrogen) was used to determine mitochondrial ROS levels. Cells were incubated for 3 h with different concentrations of metformin. A 5 m solution of MitoSOXTM Red was used to stain living cells and harvest them at 37 °C for 10 min. A flow cytometry analysis was performed after the cells had been washed with PBS.

### 4.5. Mitochondrial Membrane Potential (MMP) Analysis

Mitochondrial depolarization was measured based on a decrease in the red/green fluorescence intensity ratio. We harvested all dead and viable cells, washed them with PBS, and then incubated them at 37 °C for 15 min with 1× binding buffer containing the MMP-sensitive fluorescent dye JC-1. JC-1 fluorescence was measured using Cell Quest Pro software (BD Biosciences) on channels FL-1 and FL-2 of a FACSCalibur flow cytometer after washing the cells once in PBS. As a starting point for M2 gating, the median fluorescence intensity of the vehicle was used as the forward scatter height (FSC-H) and side scatter height (SSC-H). Their quantification of monomer (FL-1, green fluorescence) and aggregate (FL-2, red fluorescence) forms of the dye, respectively.

### 4.6. Western Blotting Analysis

U87MG, LNZ308, and LN229 cells were lysed in radioimmunoprecipitation assay buffer (150 mM NaCl, 100 mM Tris-HCl (pH 8.0), 0.1% SDS, and 1% Triton X-100) at 4 °C. Following lysate separation with sodium dodecyl sulfate-polyacrylamide gel electrophoresis, proteins were immunoblotted with antibodies against α-actinin (ACTN) (sc-17829), peroxisome proliferator-activated receptor gamma coactivator 1-alpha (PGC-1α) (sc-518052), mitochondrial transcription factor A (mtTFA) (sc-376672), Twist (sc-81417) (Santa Cruz Biotechnology, Paso Robles, CA, USA), N-cadherin (#13116), vimentin (#5741) (Cell Signaling Technology, Danvers, MA, USA), and MGMT (ab108630) (Abcam, Cambridge, UK).

### 4.7. Organotypic Slice Culture of Glioblastoma and Immunohistochemistry

Surgical samples were used to prepare slice cultures following a protocol developed by Stoppini [58,59]. To prepare the slices, we used a tissue chopper (Nickle Laboratory Engineering Co., Surrey, Surrey, BC, Canada) to slice the samples into 400 μm thick slices. We cultivated them in DMEM containing 10% FBS and 1% penicillin–streptomycin (Invitrogen, CA, USA) in the first 2 days. Then, the slices were divided into two groups, including the control and metformin treatment groups. The culture medium was changed every 2 to 3 days and fixed with 10% nonbuffered formalin for tissue processing and paraffin embedding 3 days after drug treatment. Afterward, the tissue was cut into 5 um thick sections for Ki-67 (M7240, DAKO) immunohistochemistry using the Ventana Benchmark^®^XT automated immunostainer. First, to retrieve antigens from the samples, they were heated at 125 °C for 30 min in sodium citrate buffer (0.01 M sodium citrate, pH 6.2) using a pressure cooker. As a next step, we washed each retrieval slide three times in PBS for 5 min each. We uploaded the slides to the autostainer according to the manufacturer’s instructions. After optimizing the retriever and antibody concentrations, robust staining was observed for the positive but not for the negative control. The staining density of the slides was then examined by calculating how many Ki-67-stained cells were present per mm^2^. Finally, the density of each sample was subjected to statistical analyses using Student’s *t*-test.

### 4.8. RNA Sequencing Analysis

To investigate the alteration of the transcriptomes when supplemented with metformin, we collected the LN229 cells from 12 independent experiments, including six controls and the six samples supplemented with metformin. Next, we extracted the total RNA using TRIzol reagent (Invitrogen) and assessed its quality at 260/280 nm and 260/230 nm. Then, we used the mRNA Library Kit to reverse transcribe mRNA into 300–500 bp of cDNA and added an adaptor to both ends. The adapter fragment was attached to a flow cell, amplified by Bridge PCR, and submitted to RNA sequencing on the NextSeq 550 System. Through the bioinformatics pipeline of the Precious Common Instrument Center (PCIC, https://wwwndmc.ndmctsgh.edu.tw/english) (accessed on 21 December 2020) of the National Defense Medical Center, we obtained the TPM (transcripts per million) data of each sample for further investigation. The analyses were performed with R software (R version 4.1.0, www.r-project.org, developed at Bell Laboratories, Holmdel, NJ, USA) (accessed on 21 December 2020) and relevant R packages. We used the “limma” and “ggpubr” packages to perform mRNA quantification and regression, and identify differentially expressed genes. We used an adjusted *p*-value <0.05 as the cutoff and the “heatmap” package to construct a heatmap between treatment groups.

### 4.9. Gene Set Enrichment Analysis

Gene set enrichment analysis (GSEA, https://www.gsea-msigdb.org/gsea/index.jsp) (accessed on 15 January 2021) is a valuable tool to computationally analyze gene expression data [60]. The software interprets microarray data on a gene set basis. Gene expression data from the microarray experiments are used to generate a list of genes ranked by fold change. In GSEA, genes are ranked against an identified gene set (e.g., cancer hallmarks or immune gene sets). Gene set enrichment analyses determines where genes from a gene set appear on the list of ranked genes. Each gene set has a random distribution of genes, or genes are concentrated at either end of the ranked list (top or bottom). A gene set can, therefore, be determined to be significantly enriched in a phenotypic list based on their correlation.

### 4.10. Transmission Electron Microscopy Analysis

Glioblastoma cell line LN229 was cultured in 10 cm dishes and treated with metformin, TMZ, both drugs, and the control medium in the first step. After fixation in glutaraldehyde, 2% OsO_4_ was dissolved in distilled water for 1 h, then cells were dehydrated with ethanol and embedded in EPON. We examined ultrathin sections obtained with an ultramicrotome and stained with lead citrate and uranyl acetate under a transmission electron microscope (Hitachi High-Technologies H-7500, Tokyo, Japan).

### 4.11. Preparation of Patient-Derived Organoid Culture

Briefly, after obtaining the surgical sample from a glioblastoma patient, check the tumor components (dense cellular area) and remove the adjacent part [61]. Then, microdissect the tumor into the ideal size (around 0.5–1 mm) with spring scissors or blade. Next, wash the tissue several times to remove the excess RBCs. Then, place them on the horizontal rocking shaker inside the incubator for culturing. After the first week, remove the tumor-spared pieces and keep them incubated for another 2 weeks. Later, double bisect the grown organoid (1 piece to 4 pieces) and culture for another 2 weeks. Finally, take the robust organoid sample for further experiments.

### 4.12. Statistical Analysis

A minimum of three independent experiments are conducted to obtain a mean value. We used Student’s *t*-test for all comparisons (vehicle vs. drug). A *p* value of 0.05 was set as the threshold for statistical significance.

## 5. Conclusions

Our study showed that metformin decreased metabolic activity, proliferation, migration, mitochondrial biogenesis, and mitochondrial membrane potential and increased apoptosis and ROS in some GBM cells. The sensitivity of the TMZ-resistant GBM cell line to metformin might be mediated via the suppression of mitochondrial biogenesis, EMT, and MGMT expression. Our work provides new insights into the choice of therapeutic agents in TMZ-resistant GBM treatment.

## Figures and Tables

**Table 1:** Molecular characterization of the glioma cell lines by combining our experiments and literature.

Gene	Cell line		
	U87MG	LNZ308	LN229
*IDH1*	Mutant (R132H)	WT	WT
*p53*	WT/WT	intragenic deletion loss by translocation	Codon 98 CCT(Pro)→CTT(Lys)
*ATRX*	Mutant	Mutant	Mutant
*PTEN*	splice (del. exon 3) in frame deletion	splice (del. exon 6) frameshift	WT
*p16*	del	WT	del
*p14^ARF^*	del	WT	del
*Integrated Dx*	IDH-mutant astrocytoma, Gr 4	IDH-wildtype glioblastoma, Gr 4	IDH-wildtype glioblastoma, Gr 4

## Data Availability

GSE208773 (https://www.ncbi.nlm.nih.gov/geo/query/acc.cgi?acc=GSE208773) (accessed on 15 July 2022).
